# The role of a simulator-based course in coronary angiography on performance in real life cath lab

**DOI:** 10.1186/1472-6920-14-49

**Published:** 2014-03-12

**Authors:** Ulf J Jensen, Jens Jensen, Göran Olivecrona, Gunnar Ahlberg, Bo Lagerquist, Per Tornvall

**Affiliations:** 1 Cardiology Unit, Karolinska Institutet, Department of Clinical Research and Education Södersjukhuset, Stockholm, Sweden; 2 Department of Medicine, Sundsvall-Härnösand County Hospital, Karolinska Institutet, Stockholm, Sweden; 3 Department of Coronary Heart Disease, Skåne University Hospital, Lund University, Lund, Sweden; 4 Department of Molecular Medicine and Surgery, Karolinska Institutet, Stockholm, Sweden; 5 Uppsala Clinical Research Centre, Department of Medical Sciences, Uppsala University, Sweden; 6 Department of Cardiology, Södersjukhuset, Stockholm 118 83, Sweden

**Keywords:** Learning curve, Simulator, Performance, Coronary angiography

## Abstract

**Background:**

The aim of this study was to explore if a course consisting of lectures combined with simulator training in coronary angiography (CA) could accelerate the early learning curve when performing CA on patients.

Knowledge in performing CA is included in the curriculum for the general cardiologist. The method, according to American College of Cardiology and European Society of Cardiology guidelines, for this training is not well defined but simulator training is proposed to be an option. However, the transfer effect from a CA simulator to performance in real world cath lab is not validated.

**Methods:**

Fifty-four residents without practical skills in CA completed the course and 12 continued to training in invasive cardiology. These residents were tracked in the Swedish Coronary Angiography and Angioplasty Registry and compared to a control group of 46 novel operators for evaluation of performance metrics. A total of 4472 CAs were analyzed.

**Results:**

Course participants demonstrated no consistent acceleration in the early learning curve in real world cath lab. They had longer fluoroscopy time compared to controls (median 360 seconds (IQR 245–557) vs. 289 seconds (IQR 179–468), p < 0.001). Safety measures also indicated more complications appearing at the ward, in particular when using the femoral approach (6.25% vs. 2.53%, p < 0.001).

**Conclusions:**

Since the results of this retrospective non-randomized study were negative, the role of a structured course including simulator training for skills acquisition in CA is still uncertain. Randomized transfer studies are warranted to justify further use of simulators for training in CA

## Background

According to national and international guidelines for education and training of the general cardiologist, coronary angiography (CA) experience is of high priority. However, the rationale behind these curriculums is vague since training goals for cardiologist trainees often are built on recommendations without scientific support. A log-book is a common way to register number and type of procedures performed but is limited by volume instead of quality. European Society of Cardiology (ESC) recommendations for the general cardiologist are to assist or perform 300 CAs and to interpret 1000 investigations [1]. There is also a statement that procedural skills simulators might play an important role in training invasive procedures but recommendations for how to accomplish this is lacking. The recommendation from the American College of Cardiology (ACC) is to participate in CA of at least 100 patients and for the trainee who plans to perform independent diagnostic cardiac catheterizations a minimum of 200 procedures with primary hands-on responsibilities should be performed [2].

Simulator training in CA is not well validated and transfer studies are lacking. Experts usually demonstrate a higher proficiency level in different simulator tasks and procedures as reported in several construct validation studies and the same is true for some endovascular procedures [3-7]. Most randomized controlled trials (RCTs) evaluating procedural skills achieved in virtual reality (VR) transferred to the operating room (OR) have explored surgical procedures such as laparoscopy and flexible endoscopy [8-10]. A common feature of these RCTs are that they are few and conducted with a rather limited number of participants as concluded in several systematic reviews [11-13]. None of the RCTs have had patient outcome as an end-point which should be the ultimate criterion for the quality of the training programs using procedural skills simulators. A review by Lynagh et al. aimed for evaluating the effectiveness of medical skill laboratories or procedural skills simulators [14]. Twelve of the included studies assessed the transfer of simulator performance to clinical skills performance on real patients but none on endovascular procedures. The conclusion drawn from this review was that medical skills laboratories do lead to improvement compared to standard training when transferred to real life but that there is a lack of well-designed trials. A Cochrane review by Walsh et al. concluded that there is insufficient evidence to advise for or against the use of VR training, this time regarding gastrointestinal endoscopy [15]. A recent review and meta-analysis including all simulator training environments from practical skills training to team training has been conducted as an attempt to justify Simulation-Based Medical Education (SBME) as a concept for the future of medical training [16]. This meta-analysis of technology-enhanced simulation by Cook et al. identified 609 studies of which 137 were randomized [16]. Only 10 studies explored the training effect in endovascular procedures. The overall conclusion was that knowledge, time skills and general behavior were favored by simulator training but no sub-group analysis was performed regarding procedural skills in endovascular procedures. When exploring the transfer effect from endovascular simulators to real patients a review article by Tsang et al. included three studies on carotid stenting and four on peripheral vascular angioplasty [17]. Only one of the RCT’s showed transferability from VR to OR and that was in peripheral vascular intervention [18]. In a recently published review about the future of simulation technologies for complex cardiovascular procedures references were made to several VR validation studies in endovascular procedures; however all of those studies used animal models for validation [19] and to our knowledge, no study has evaluated the transfer of CA skills from VR to OR in humans.

The aim of this study was to evaluate if a structured training program including simulator training could improve the early learning curve for trainees in CA and thus make the learning process safer for the patient (transfer validity).

## Methods

### Course

The course was founded and initiated by the authors in 2006 and only minor changes have been made over the years. The course was recommended by the Swedish Society of Cardiology and the Swedish Heart Association. At all course events residents had access to two simulators and three instructors. Each course was limited to six participants in order to keep a high exposure to the simulators. Three instructors, all experienced invasive cardiologists, were responsible for proctoring and lectures. A total of six hours of dyad proctored simulator training and six hours of theoretical lectures were completed during two days. Course participants aiming for certification in CA and living in proximity to the training center had an opportunity to obtain further solo VR experience and to perform a practical examination on the simulator. A goal of obtaining certification was not compulsory for course participation.

The goal of VR training was to obtain a safe behavior of the procedure completing CA with a small but sufficient amount of contrast used and accurate virtual C-arm angulations to project the coronary vessels in recommended views. Instructions of how to handle fluoroscopy, wires and catheters safely during CA were also given. In addition, femoral arterial puncture technique was practiced on a dummy with “subcutaneous” arterial-like rubber tubes constructed to give pulsatile backflow of artificial blood when entering the vessel. Puncture technique ad modum Seldinger was demonstrated by the tutors in a stepwise fashion to ensure that all parts were accomplished in a correct way. The course participants had subsequent dyad practice in arterial puncture technique for an average of 90 minutes.

The theoretical part of the course included lectures about the CA procedure regarding anatomy, pharmacology, complications, puncture technique, radiation safety and materials, in total six hours. A web-based theoretical course was offered as a complement to live lectures during the two last years.

### Study subjects

The course participants were all senior residents in cardiology and in their second half of their four years of training. They were recruited from all geographical areas of Sweden to attend the course by advertisement in the journal of Swedish Society of Cardiology and by direct mail to all cardiology units and invasive centers in Sweden. During six years, 54 residents participated and completed the course at two different sites in Sweden. Twelve of the course participants progressed to become invasive cardiologists. Five of these participants had free optional additional training in the simulator to enable examination and certification.

### Simulator

The two centers involved in the course had each access to one VR simulator (Mentice VIST™) on a dedicated center for simulation (Clinical training center, Karolinska University Hospital, Stockholm and Practicum, Skåne University Hospital, Lund). During the course an additional identical simulator was borrowed from one company involved in the device industry. Mentice VIST™, Gothenburg, Sweden is a VR simulator where you can practice coronary angiography in full scale, using real catheters and wires modified to fit the machine. The virtual femoral arterial access was premade. Potentially harmful parts in the investigation such as radiation, fluoroscopy and filming were simulated as well as contrast injection.

### Study protocol

The design of the study was a retrospective cohort study where the cohort were residents exposed to the course progressing to invasive cardiologists and the controls novel operators found in the Swedish Coronary Angiography and Angioplasty Registry (SCAAR). All hospitals in Sweden performing CA (n = 30) and interventions (n = 29) register all their procedures. The definition of a beginner was set to be an invasive cardiologist who started to perform CA between 2005 and Q1 2012 and had performed at least 80 CAs and at least 40 CAs annually. A total of 58 novel CA operators were identified in Sweden during the observation period of seven years. Twenty percent attended the course. Cohort (n = 12) and controls (n = 46) were tracked in the SCAAR registry and met the inclusion criteria for beginners. No other practical simulator-based courses in CA were held in Sweden during the observation time and the likelihood for controls to be simulator trained in CA was low. There were no gender differences between the two groups (16.6% vs. 17.3% females). Study metrics representing proficiency in CA have been previously described and these were compared between groups [20]. Complications during CA is associated to proficiency and during training most often related to the access site with increased risk of bleeding when using the femoral approach. The number of complications was therefore analyzed also in relation to access site. Elapsed time from course completion to performing the first CA or previous CA experience might have an impact on the real life performance and was therefore also explored.

### Statistical analysis

Data are presented as median and inter-quartile range (IQR), mean ± SD or median (range) and numbers (%). Descriptive summary statistics were used where appropriate. Differences were tested with Mann–Whitney *U*-test or Chi-Square test. Kruskal-Wallis was used where appropriate. Analyses were performed using Statistica version 10, (Statsoft, Inc, Tulsa, OK, USA)

### Ethics

All participants received written information about the project. The protocols and procedures were approved by the local ethics committee for human research at Karolinska Institutet and at Uppsala University ref.nr. 04-202/1. The studies were performed according to the declaration of Helsinki and good clinical practice. Informed consent was provided by all participating residents and consultants. Analyzing retrospective CA procedural data in the SCAAR registry was covered by a general approval from the ethics committee at Uppsala University.

## Results

**Figure 1:**
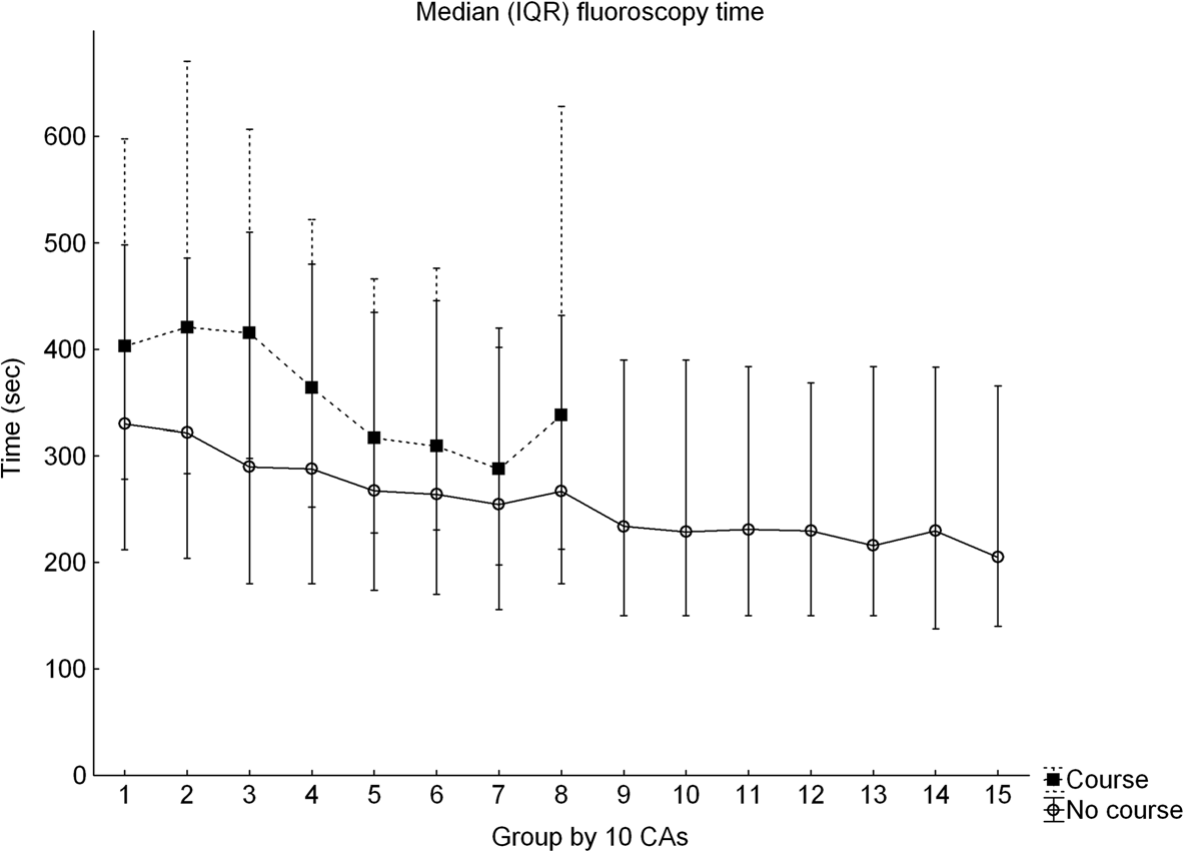
**Median fluoroscopy time for course participants and controls representing the early learning curve.** CAs = coronary angiographies. Values in median (IQR).

**Figure 2:**
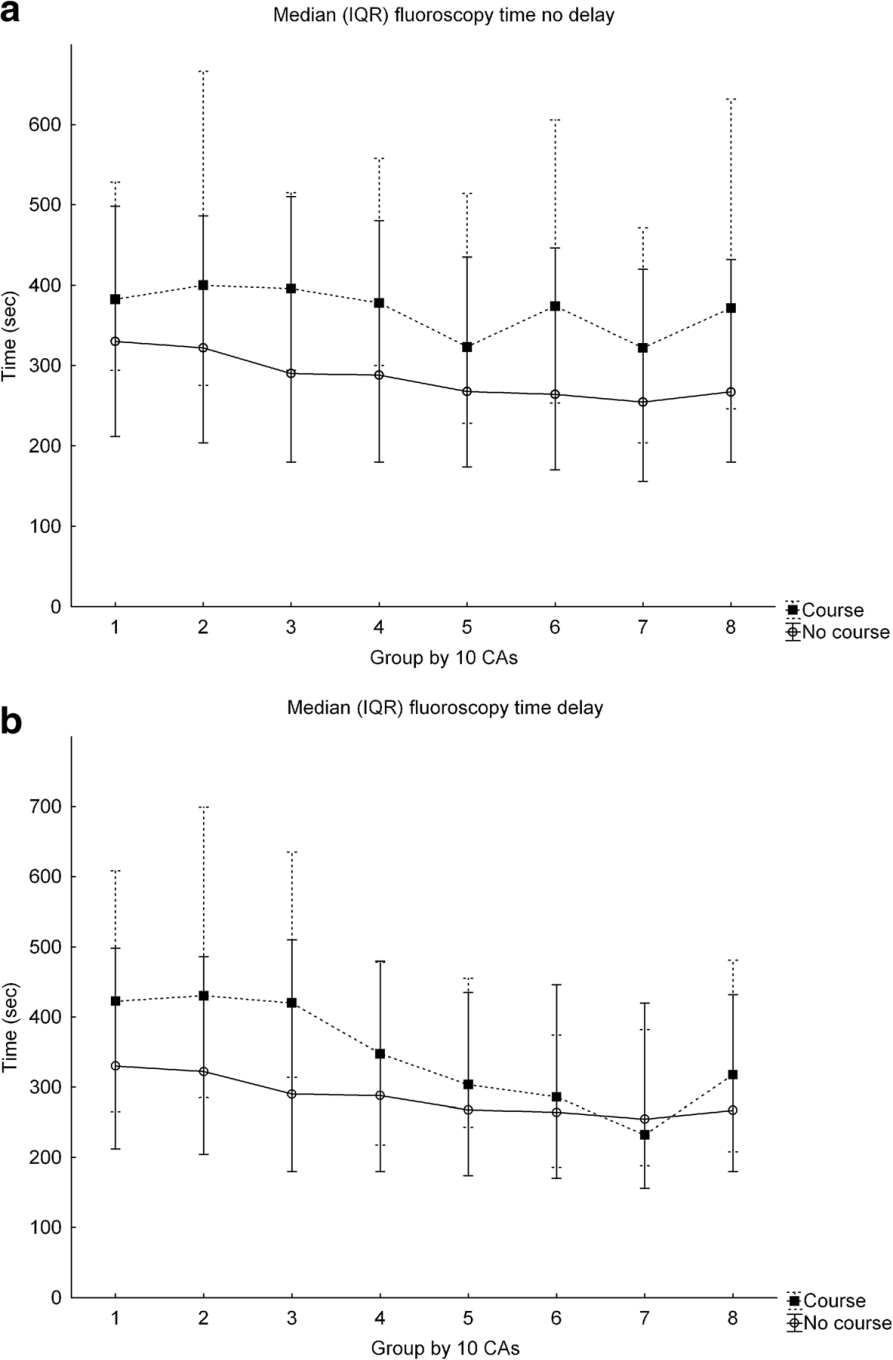
**Median fluoroscopy time course participants and controls representing the early learning curve a) without delay and b) with delay from course to first CA.** CAs = coronary angiographies. Values in median (IQR).

**Figure 3:**
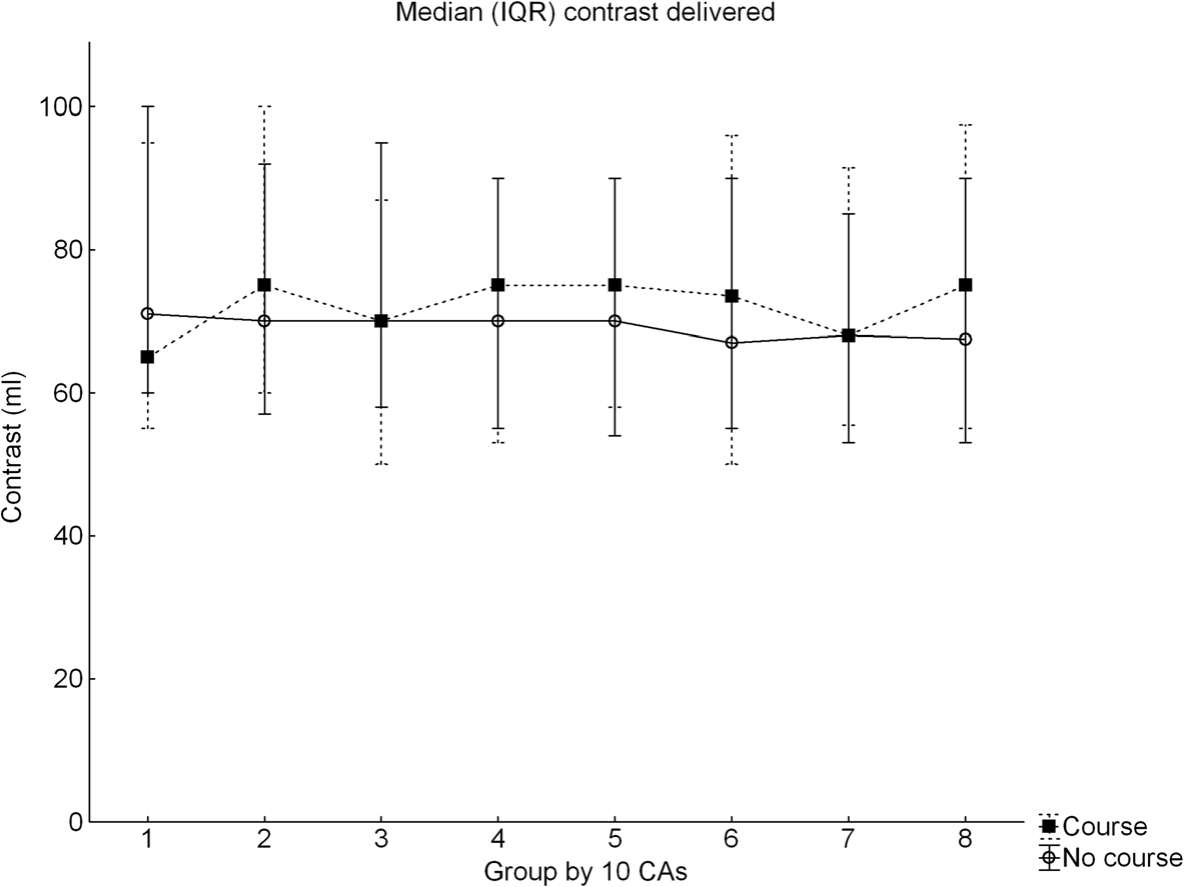
**Median contrast delivery course participants and controls.** CAs = coronary angiographies. Values in median (IQR).

A total of 4472 CAs were analyzed in the SCAAR registry. In the metrics extracted from SCAAR and representing proficiency in cath lab, the trainees completing the course performed worse regarding fluoroscopy time compared to the controls which in turn demonstrated a typical learning curve showing reduction of fluoroscopy time over time (Figure 1) [20,21]. Course trainee fluoroscopy time was in median 360 seconds (IQR 245–557) vs. 289 seconds (IQR 179–468), p < 0.001, in non-course trainees (Table 1). Course participants demonstrated a less consistent improvement and the learning curves between the groups did not persistently cross during the observation time. The pattern was the same independent of prior experience or lapsed time between taking the course and performing the first CA (Figure 2a-b), (Table 2). No learning curve was demonstrated in the use of contrast and the groups used the same amounts (Figure 3). There were no differences in the rate of complications at the cath lab, 0.57% vs. 0.92%, p = 0.421 but the course participants had more complications appearing at the ward, in particular, when using the femoral approach, 6.25% vs. 2.53%, p < 0.001 (Table 3). The total time for the procedure is not registered in SCAAR and could therefore not be evaluated.

**Table 1:** Metrics course participants to control

**Metric**	**Course – [46]**	**Course + [12]**	**p-value**
Contrast total	70 (55–90)	70 (55–95)	0.687
Fluoro CA 1-10	329 (212–498)	430 (278–597)	0.001
Fluoro CA 11-20	322 (203–486)	420 (283–670)	<0.001
Fluoro CA 21-30	289 (179–510)	415 (297–606)	<0.001
Fluoro CA 31-40	287 (179–480)	364 (252–522)	<0.001
Fluoro CA 41-50	267 (174–435)	317 (228–466)	0.026
Fluoro CA 51-60	264 (170–445)	309 (230–476)	0.018
Fluoro CA 61-70	254 (156–419)	287 (197–402)	0.049
Fluoro CA 71-80	267 (179–432)	337 (212–627)	<0.001
Fluoro CA 1-80	289 (179–468)	360 (245–557)	<0.001

Metrics course participants to control. Median values in seconds, (IQR). [# case or control]. Fluoro = fluoroscopy. + indicates course participants. – indicates controls. Mann–Whitney *U* test. CA = coronary angiography.

**Table 2:** Baseline experience at course participation or delay to first CA

**Participant**	**Course**	**First CA**	**Experience/time - angio**
1	060914*	080110	69 weeks
2	060914	090907	155 weeks
3	071119*	071023	17 CA
4	080911	080228	27 CA
5	080911*	110617	144 weeks
6	081211	090114	5 weeks
7	081211	090219	9 weeks
8	081211	090309	12 weeks
9	091126	090908	31 CA
10	091126*	090915	33 CA
11	091126	090212	35 CA
12	100610*	111128	77 weeks

Baseline experience at course participation or delay to first CA. *marks participants with additional sim-training. CA = coronary angiography.

**Table 3:** Complications during the first 80 procedures

**Course**	**Lab comp**	**Ward comp**	**Fem lab comp**	**Rad lab comp**	**Fem ward comp**	**Rad ward comp**
+ [12]	5/878 (0.57)	38/878 (4.33)	3/528 (0.57)	2/350 (0.57)	33/528 (6.25)	5/350 (1.43)
- [46]	33/3594 (0.92)	67/3594 (1.86)	19/1973 (0.96)	14/1620 (0.86)	50/1973 (2.53)	17/1620 (1.05)
Total	38	105*	22	16	83*	22

Complications during the first 80 procedures, (%). [participants]. * = p < 0.001 tested by Chi-Square. Comp = complication, Fem = femoral, Rad = radial. + indicates course participants. – indicates controls.

## Discussion

In this study, the simulator-based course did not result in an acceleration of the learning curve, instead course participants had longer fluoroscopy time than controls, moreover time from course event or previous CA experience did not affect the performance metrics. The number of complications in the course participants was elevated compared to the control group suggesting that a structured course including simulator training might have a negative impact on the learning process of CA.

### Transferability

Simulation experience in medical procedures is regarded as the future for medical education and training. However, convincing data of transferability from VR to OR in endovascular procedures have been shown only for a few procedures. Berry et al. showed a transferability from VR to a OR in a pig model of iliac vascular intervention and De Ponti et al. concluded that VR training in cardiac transseptal puncture (TSP) resulted in a shorter training time, a higher assessment score and fewer errors during TSP in patients [22,23]. No data have been published regarding VR transfer effect to CA.

### Aim and results

In this study, our aim was to evaluate if a course in CA using simulators could accelerate the early learning curve in performing CA on real patients and if the patient benefited from this preparatory VR training. The results demonstrated that using simulators as a learning tool to increase clinical skills is not convincingly obvious and that the training actually can impair the early learning curve and result in worse outcome in patients.

Only one previous study has reported an impaired performance in a VR trained group and that in a non-endovascular procedure [24]. In our study, residents taking a course in CA, including theoretical and practical training, actually performed worse in parameters previously demonstrated representing proficiency [20]. Course participants used longer fluoroscopy time and had more complications when using the femoral access. The initial learning curve was not altered whether they had a delay from the course or not to CA or had some CA experience when taking the course assuming that not even a practical course in close proximity to performing the procedure in real life had any positive effect on performance.

### Potential detrimental effect of VR training

Unfortunately, this study could not demonstrate a transfer effect from VR to OR in this setting. One possible reason for that is that despite proctored VR training the actual simulator training was not structured in that sense that no benchmarked proficiency level was reached before performing CA on patients. Proficiency-based pretest training has been a common denominator in VR studies demonstrating transferability and proposed to be the paradigm shift in VR skills training [9,25]. However, by the time for these simulator-based CA courses the expert proficiency level in the simulator was not known. One might argue that a longer course or training period would have amplified the skills achieved in the simulator resulting in a higher proficiency level in cath lab but on the other hand this would have been time-consuming for the trainee as well as for the proctors. A second hypothesis for the failure to show a transfer benefit from VR training to cath lab might be that the trainee becomes too self-confident after VR training thereby asking for less assistance with the actual CA procedure. In contrast the mental preparation for the procedure that simulator training offers might be beneficial. Mental imaginary of completing a stressful task has a potential to prepare the performer for possible instrument handling difficulties and hazards and subsequent mental stress. However, a randomized study exploring whether mental imaginary of a surgical procedure could improve the performance failed to show any benefit in analogy with this preparatory simulator training [26]. The course attendees had longer fluoroscopy time, perhaps dependent of time spent on unnecessary handling of catheters something not dangerous to the VR patient. However, extended catheter handling in patients is correlated to catheter-related emboli and must be prevented [27]. Supervised stepwise training in cath lab like the old master-apprentice model with graded increased responsibility is perhaps as safe as simulator training and more cost efficient. Zendejas et al. [28] made an attempt to systematical review cost as an outcome of SBME. Fifteen studies were indentified comparing simulation training to other instructional modalities but none reported a formal cost-effectiveness analysis. Discussions promoting SMBE usually compare the cost of SBME to a hypothetical medical error thus saving money. However, in this study course participants actually had more complications than the control group using the femoral approach indicating a higher cost.

### Reducing procedural complications

The types of complications were not classified but bleeding associated to the puncture site is likely to be the major part. Puncture of the access site is not achievable in the VIST simulator instead the course participants received arterial puncture training on a dummy. This training modality is not validated in a transfer setting and might therefore be of no use or even harmful. To overcome this problem one might argue that the arterial puncture training simulator must consist of a more arterial tissue-like texture and be validated in a proficiency-guided randomized transfer environment. To optimize training conditions in a procedural skills simulator like VIST and to promote an ultimate training effect transferring to real life some circumstances are likely to increase this effect: First, proficiency guided training, i.e. well-defined training goals in the simulator in different quality metrics known to affect patient outcome established by experienced CA operators. Second, quality and not time-dependent training should be performed to ensure a high lowest threshold for passing the training course. Finally, since access site complications are more common during training, a validated arterial puncture procedural training facility, dummy or simulator, must be added to the course curriculum to enable a safe procedure from start to end.

A randomized single-blinded VR transfer study in CA based on the above arguments is currently running in Stockholm and will be completed early 2014 and hopefully the future role for VR training in CA will be clearer.

### Study limitations

The limitations in this study were several. It was not randomized and thereby not adjusted for potential confounders such as poor performance of the course participants. Course attendees progressing to invasive cardiology were few but corresponded to 20% of all novel operators in Sweden over the observation time of seven years. The course was short, only including 6 hours of simulator training, but the trainees were practicing in pairs known to increase the learning process and the sessions were proctored by experienced invasive cardiologists providing proximate feedback ensuring appropriate catheter behavior. Simulator training sessions were not guided to reach an expert level since this level was not known at the time of the courses. All participants did not advance straight to the cath lab doing CAs after completion of the course. Five of the course participants had some experience of performing CA and they all continued with CAs in direct proximity to the course without delay (Table 1). However, delay or experience did not seem to affect the early learning curve (Figure 2a-b).

The study is based on a well-validated registry, tracking all coronary interventions in Sweden. However, this registry contains limited information regarding the operator. For example, we do not know how much help or supervision the trainees had during their initial procedures in cath lab. Another limitation might be that only one procedural parameter was used for demonstrating competency but a previous registry study explored several such parameters and only fluoroscopy time demonstrated a true learning curve and an association to patient outcome [20]. Total time for a CA was not recorded and the amount of radiation during a CA was not possible to measure since it is not comparable between different sites because of different cath labs. These metrics is by all means important and might possibly represent proficiency level but could not be tested. However the strength of this study is that it is multicenter excluding site bias and representing the real world situation.

## Conclusions

Since the results of this retrospective non-randomized study were negative, the use of simulators is not necessarily associated with improved learning of CA. In this study, the concept of cognitive and practical training without a well-defined training goal resulted in an impaired learning curve and worse performance in real life cath lab. Randomized transfer validation studies with well defined expert training goals are warranted to justify further use of simulators for CA training.

## Abbreviations

ACC: American College of Cardiology; CA: Coronary Angiography; ESC: European Society of Cardiology; IQR: Inter Quartile Range; OR: Operation Room; PCI: Percutaneous Coronary Intervention; SCAAR: Swedish Coronary Angiography and Angioplasty Registry; SD: Standard Deviation; SBME: Simulation-Based Medical Education; TSP: Transseptal Puncture; VR: Virtual Reality; VIST™: Vascular Intervention Simulation Trainer.

## Competing interests

The authors of this manuscript declare that no financial or non-financial competing interests exist related to the content of the manuscript. None of the authors have received reimbursements, fees, funding or salary from an organization that may gain or lose financially from the publication. None of the authors hold any stocks or shares or are involved in any patents relating to the content of the manuscript.

## Authors’ contributions

UJ: The primary author responsible for the completion of the first draft of the manuscript and the revision. Acted as one of two teachers and proctors of the courses and the designer of the practical and theoretical course. Made the final statistical calculations. JJ: Was a contributor to the design of the course. Was also a reviewer of the manuscript before submission. GO: Participated as one of two course instructors and a contributor to the content and proctoring during the courses. Was also a reviewer of the manuscript before submission. GA: Participated as a contributor to the design of the study based on a vast experience in simulator technology and practical teaching. Was also a reviewer of the manuscript before submission. BL: Responsible for the data collecting from the national registry (SCAAR) and for the initial statistical calculations. Was also a reviewer of the manuscript before submission. PT: Together with UJ responsible for the overall course and study design and the major reviewer in the completion of the manuscript before submission. All authors read and approved the final manuscript.

## Pre-publication history

The pre-publication history for this paper can be accessed here:

http://www.biomedcentral.com/1472-6920/14/49/prepub
